# Reliable gene expression profiling of formalin-fixed paraffin-embedded breast cancer tissue (FFPE) using cDNA-mediated annealing, extension, selection, and ligation whole-genome (DASL WG) assay

**DOI:** 10.1186/s12920-016-0215-4

**Published:** 2016-08-20

**Authors:** Mahesh Iddawela, Oscar M. Rueda, Marcus Klarqvist, Stefan Graf, Helena M. Earl, Carlos Caldas

**Affiliations:** 1 Cancer Research UK Cambridge Institute, Li Ka Shing Centre, Robinson Way, Cambridge, CB2 0RE UK; 2 Department of Oncology, Addenbrooke’s Hospital, University of Cambridge, Hills Road, Cambridge, CB1 9RN UK; 3 Cambridge Breast Unit, Addenbrooke’s Hospital, Cambridge University Hospitals NHS Foundation Trust, Cambridge, UK; 4 NIHR Cambridge Biomedical Research Centre and Cambridge Experimental Cancer Medicine Centre, Cambridge, UK; 5 Department of Anatomy and Developmental Biology, Monash University, Clayton, Victoria 3800 Australia; 6 School of Clinical Sciences, Monash University, Clayton, Victoria Australia

**Keywords:** FFPE, WG DASL, Molecular Markers, Breast Cancer

## Abstract

**Background:**

The difficulties in using formalin-fixed and paraffin-embedded (FFPE) tumour specimens for molecular marker studies have hampered progress in translational cancer research. The cDNA-mediated, annealing, selection, extension, and ligation (DASL) assay is a platform for gene expression profiling from FFPE tissue and hence could allow analysis of large collections of tissue with associated clinical data from existing archives, therefore facilitating the development of novel biomarkers.

**Method:**

RNA isolated from matched fresh frozen (FF) and FFPE cancer specimens was profiled using both the DASL whole-genome (WG) platform, and Illumina BeadArray’s, and results were compared. Samples utilized were obtained from the breast cancer tumour bank held at the Cambridge University Hospitals NHS Foundation Trust.

**Results:**

The number of reliably detected probes was comparable between the DASL and BeadArray platforms, indicating that the source of RNA did not result in a significant difference in the detection rates (Mean probes- 17114 in FFPE & 17400 in FF). There was a significant degree of correlation between replicates within the FF and FFPE sample sets (*r*^2^ = 0.96–0.98) as well as between the two platforms (DASL vs. BeadArray *r*^2^ = range 0.83–0.89). Hierarchical clustering using the most informative probes showed that replicate and matched samples were grouped into the same sub-cluster, regardless of whether RNA was derived from FF or FFPE tissue.

**Conclusion:**

Both FF and FFPE material generated reproducible gene expression profiles, although there was more noise in profiles from FFPE specimens. We have shown that the DASL WG platform is suitable for profiling formalin-fixed paraffin-embedded samples, but robust bioinformatics analysis is required.

## Background

Formalin-fixed paraffin-embedded (FFPE) samples are a valuable source of clinical material, with great potential for predictive and prognostic biomarker discovery. RNA extracted from FFPE tissue is degraded, and therefore analysis is difficult since formalin fixing leads to cross-links with proteins and mono-methanol modification of nucleotide bases [1–3]. Improvements in technology has made analysis of this material feasible, with considerable potential for translational research using FFPE tissue.

The cDNA-mediated, annealing, selection, extension, and ligation assay (DASL®, Illumina, San Diego) is a platform that was developed for high-throughput gene expression analysis of partially degraded RNA. This assay initially assessed the expression of up to 1536 probes (a maximum of 512 genes, with three probes per gene) using 100–200 ng of degraded RNA [4]. A combination of random hexamers and oligo-dT priming is used for cDNA synthesis. The assay is designed to analyse 50 bp RNA fragments, and the sensitivity of the platform is enhanced by PCR amplification using common primers and by having 30 replicate beads per probe. These arrays are assembled randomly, and the position of each probe is determined following manufacture, which reduces spatial effects [5].

The initial DASL report showed that reproducible and disease-specific gene expression profiles can be generated using FFPE samples up to 10 years old [6]. They also compared gene expression in fresh frozen (FF) and FFPE specimens using eight matched cancer and normal samples, and derived a signature that could differentiate between cancerous and normal tissue in breast and colorectal cancers.

One of the limitations of the original DASL assay was the limited number of transcripts interrogated. Hence, the introduction of the whole-genome DASL assay (DASL WG), which profiles 24,000 transcripts, represents a marked improvement. In the initial report [7], the number and overlap of probes in FFPE samples compared to matched FF specimens were 88 % and 95 %, respectively. Furthermore, there was a 74 % overlap between differentially expressed genes in FF and FFPE samples. The assay was able to detect 1.5–2-fold changes in gene expression in FFPE specimens and produced high self-correlation between replicates (r^2^ > 0.98). Chien et al. showed that the DASL WG assay can be used to generate a signature typical of low-grade serous ovarian carcinoma and thereby distinguish it from borderline tumours [8]. This was subsequently used to differentiate serous and borderline tumours in three independent datasets with 80 % sensitivity. Another study investigated the effectiveness of DASL WG in predicting subtypes of familial breast cancer from FFPE samples; however the study did not include a direct comparison with matched FF material [9]. In another study, comparison of expression profiles between FFPE and corresponding normal FF tissues found that only one quarter of genes identified in FF samples were also detected in FFPE samples [10]. Several studies using DASL have been published, but the majority have not used matched FF and FFPE samples, or have used different platforms to analyse FF samples [11, 12].

Here, we report a robust assessment of the DASL WG platform in gene expression profiling of matched FFPE and FF RNA samples from breast cancers. For orthogonal comparison, the same FF samples were also profiled with the Illumina BeadArray platform.

## Methods

Matched FF and FFPE samples from a cohort of breast cancer patients treated in 1999 at Cambridge University Hospitals NHS Foundation Trust were obtained from the tumour bank. This study has been approved by the Cambridge University Hospital Research Ethics committee.

### RNA extraction

Five to eight 5 μm-thick sections were cut from each block of FFPE tissue, and the samples were deparaffinised with xylene and digested with proteinase K for 14 h. Other steps, including purification and DNase treatment, were performed according to the manufacturer’s protocol, and total RNA was stored at −80 °C after extraction (Roche high Pure Kit). The FF RNA was extracted using the Qiagen mirNEasy kit (Qiagen) according to the protocol.

### Whole-genome DASL

Total RNA extracted from FFPE and FF samples was converted to cDNA using oligo-dT_18_ and random nanomer primers. Pairs of query oligonucleotides, including three unique pairs for each of 24,000 genes, were annealed to complimentary sequences (~50 bp) flanking the specific cDNA target site. The biotinylated cDNA was then bound to streptavidin particles, and mis- and non-hybridised oligonucleotides were washed away. Each primer had a gene-specific sequence and a universal PCR primer site; a prolonged annealing time of 16 h was used, with a temperature gradient from 70 to 30 °C. PCR was performed using universal primers and a single colour (Cy3) assay. The amplified cDNA was then denatured with NaOH, precipitated, and washed with 75 % alcohol. The cDNA was then re-suspended and hybridised to Illumina Human Reference 8 BeadArrays for 16 h at 65 °C. The slides were washed and imaged using the Illumina BeadArray Reader (Illumina, San Diego, CA).

### Illumina expression BeadArrays

Total RNA (200 ng) from the same FF tumours assessed in the DASL WG assay was used to generate cRNA, using the Illumina TotalPrep RNA amplification kit (Ambion). Reverse transcription with the T7 oligo (dT) primer was used to generate first strand cDNA, from which a second strand was produced using DNA polymerase. The RNA was subsequently degraded with RNase H and cleaned-up. *In vitro* transcription and biotin-labelled UTP were used to generate multiple copies of biotinylated cRNA, which was purified with a filter cartridge and quantified using a NanoDrop spectrophotometer.

The labelled cRNA target (1.5 μg) was hybridised to an array according to the Illumina HumanRef-6 BeadArray protocol. A maximum of 10 μl of cRNA was mixed with 20 μl of GEX-HYB hybridisation solution. The pre-heated 30 μl sample was dispensed into the large sample port of each array and incubated for 18 h at 58 °C at a rocker speed of five. The samples were hybridised to Illumina BeadArrays (Human Ref 6) and were washed according to the standard protocol and scanned with a BeadArray Reader (Illumina, San Diego, CA).

### Analysis of gene expression data

Data from the Illumina microarray experiments were analysed using BeadStudio® software (Illumina, San Diego) and R (version 2.6.0) [13].

During this analysis, image files were loaded using the BeadArray package. The BASH algorithm was used to remove spatial artifacts [14]. The algorithm generates a grid of neighbours for each bead and identifies three types of defects: compact, diffuse (regions that contain more outliers than expected by chance), and extended. The average intensities of each probe were computed to summarise replicates and finally the data was quantile normalised.

Data for both experiments was combined to give a total of 24526 probes. Comparisons between FF (BeadArray) and FF (WG DASL) and between FF (WG DASL) and FFPE (WG DASL) were performed using identical probes with Student’s t-tests. For carrying out comparisons between frozen samples used in DASL and BeadArray, a linear model per probe was fitted to detect those showing substantial differences. All probes with a *p*-value smaller than 0.05 were selected for clustering analysis.

For comparisons between paraffin and frozen samples in the DASL WG assay, a similar linear model was fitted and the probes with *p*-value < 0.05 were selected.

Correlations between these types of samples were computed using all probes and this selection of good probes. The final subset of probes that were selected in the platform and the tissue comparisons were used as input for the final cluster analysis. The data is deposited in a public data repository.

## Results

### Samples and quality control

As part of this study, five matched FFPE and FF samples were profiled in multiple technical replicates using the DASL WG assay. The eight FF cancers were also profiled using Illumina BeadArray for inter-platform comparison, including five described above (Table 1). The size of the recovered RNA ranged between 100 and 200 base pairs in length. *RPLP13* QRT-PCR provided a quality control measure and only samples with a cycle threshold (Ct) value of 22–26 were deemed suitable for further analysis.

**Table 1:** Correlation of gene expression between matched FFPE and FF samples pre- and post-gene selection using the DASL WG assay and Illumina BeadArray

	Before gene selection	After gene selection
DASL WG FFPE vs. FF	r^2^	r^2^
99037	0.83	0.96
99085	0.86	0.97
99025	0.86	0.98
99028	0.85	0.96
99066	0.89	0.98
DASL WG FFPE vs. FF BeadArray		
99037	0.80	0.83
99085	0.82	0.86
99025	0.81	0.86
99028	0.81	0.85
99066	0.81	0.89

## Assay performance

**Fig. 1:**
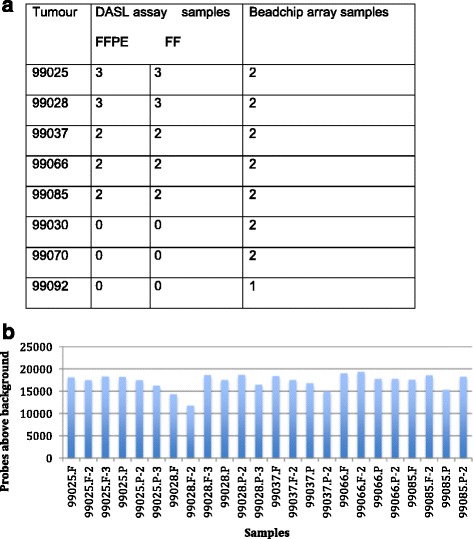
**a** Samples and the number of technical replicates used for the DASL WG and Illumina BeadArray analysis & (**b**) The number of probes detected above background from Fresh (F) and FFPE (P) samples in the DASL WG assay

To assess the DASL assay performance, the average number of genes detected above background were compared in the five matched FF and FFPE samples. The mean number of probes detected above background in FFPE samples were 17114 (range 15291–18662) and 17400 (range 11763–19320) in FF samples, and more probes were detected reliably in four of the FF samples used (Fig. 1b). The mean number of probes detected in replicates of each sample was 17598 (range 14872–19146) for FF and 17053 (16769–17773) for FFPE.

The probes were carefully re-annotated according to alignment characteristics (perfect match, good, bad or no match) and the signal was compared against these features to quantify their effect. While there was a significant low signal in the no match probes, the perfect matched probes had the greatest signal as expected. When the reliability of probe detection and the size of the genes were investigated, there was a significant difference. The genes that were shorter were more lilkely to be reliable than those which are longer. Further, the proportion of detected probes in FF and FFPE samples were compared, prior to normalisation, to assess the quality of the signal in the DASL assay. This showed that as expected, there is a greater variation in the signal intensity in the FFPE samples compared to FF samples.

The reproducibility of the WG DASL assay for FFPE (using hierarchical clustering analysis), showed that all replicate FFPE samples clustered together (except for 99028P-3, which is a single outlier), suggesting that the assay has a high degree of reproducibility for FFPE analysis.

## FFPE vs. FF sample analysis

**Fig. 2:**
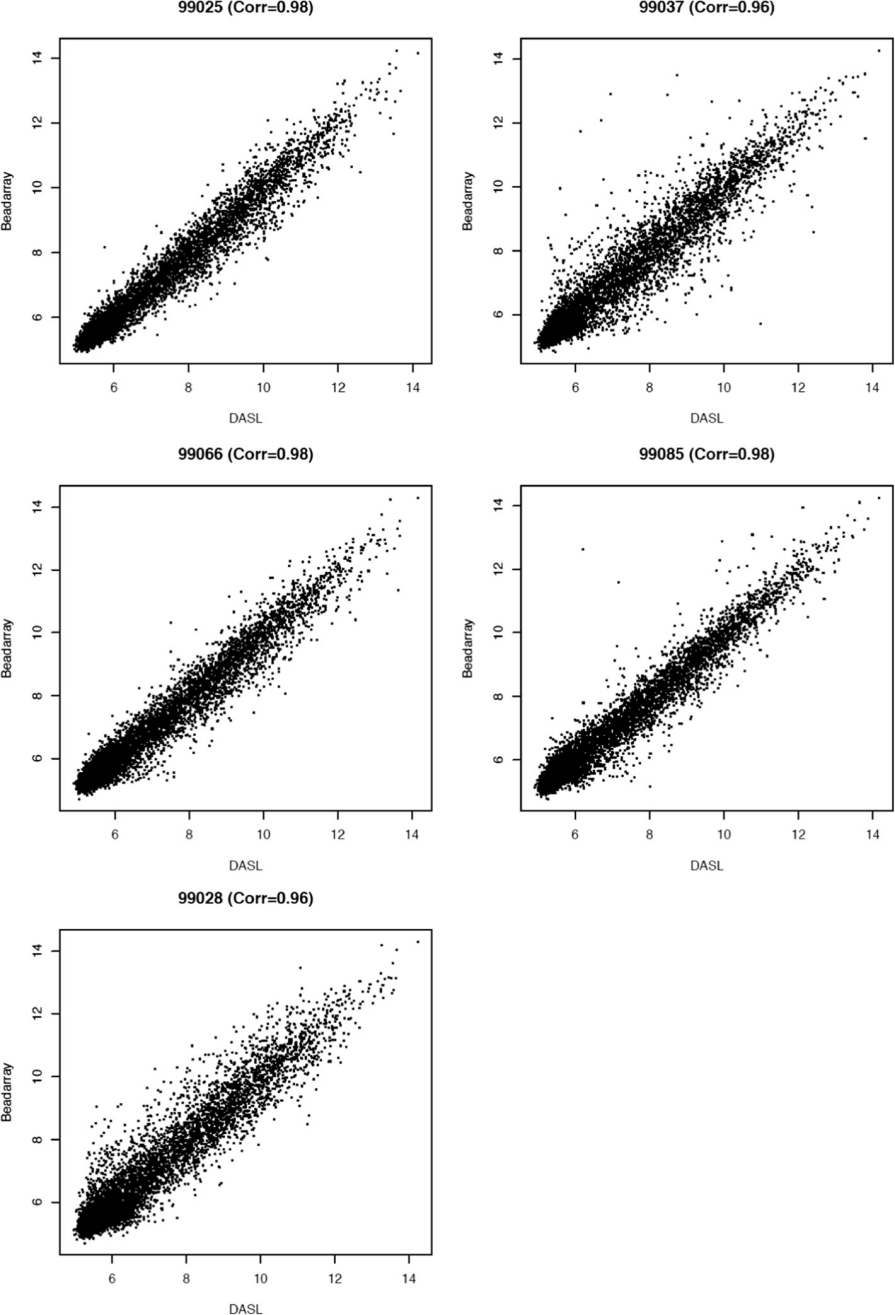
Scatter plots showing correlations of gene expression between FF vs. FFPE samples assessed using the DASL WG assay, following selection of the best performing probes

In order to assess the robustness of the DASL WG platform for analysing FF and FFPE samples, the correlation between different sample types was computed. It ranged between 0.83 and 0.89 for all the probes, and improved to 0.96–0.98 upon gene selection undertaken as described in the methods section (Figs. 1 and 2, Table 1).

**Fig. 3:**
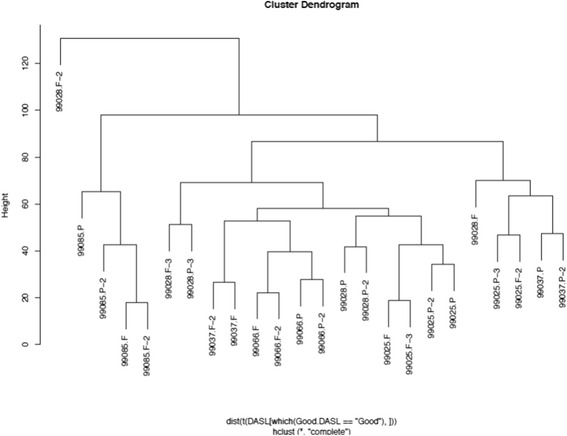
Hierachial clustering of samples assessed using DASL WG assays. FFPE & FF samples assessed using the DASL WG assay clustered together in the majority of samples (note 99028 F is an outlier as it had very few probes detected)

Hierarchical clustering using the best performing probes showed significant homogeneity, with profiles from four of the samples (99085, 99028, 99066 and 99025) clustering together irrespectively of the origin (Fig. 3). There were outliers, but these were usually samples with a lower number of genes detected reliably.

### DASL WG assay and Illumina BeadArray

The correlation between all the probes in the DASL WG and BeadArray was assessed to compare the two platforms. The correlation ranged between 0.80 and 0.82, and improved to 0.83–0.89 when only the best performing probes were used for the analysis (Table 1).

Although the DASL WG assay data (using FFPE and FF samples) and the BeadArray (using only FF specimens) are two independent platforms, hierarchial clustering using all probes showed that samples grouped according to the RNA sample source and the array platform used. In fact, all FFPE samples were closer together in the hierachial clustering tree compared to FF specimens assessed with the BeadArray. Interestingly, the FF samples assessed by Illumina BeadArray and DASL WG did not cluster next to each other, but rather in two distinct groups.

**Fig. 4:**
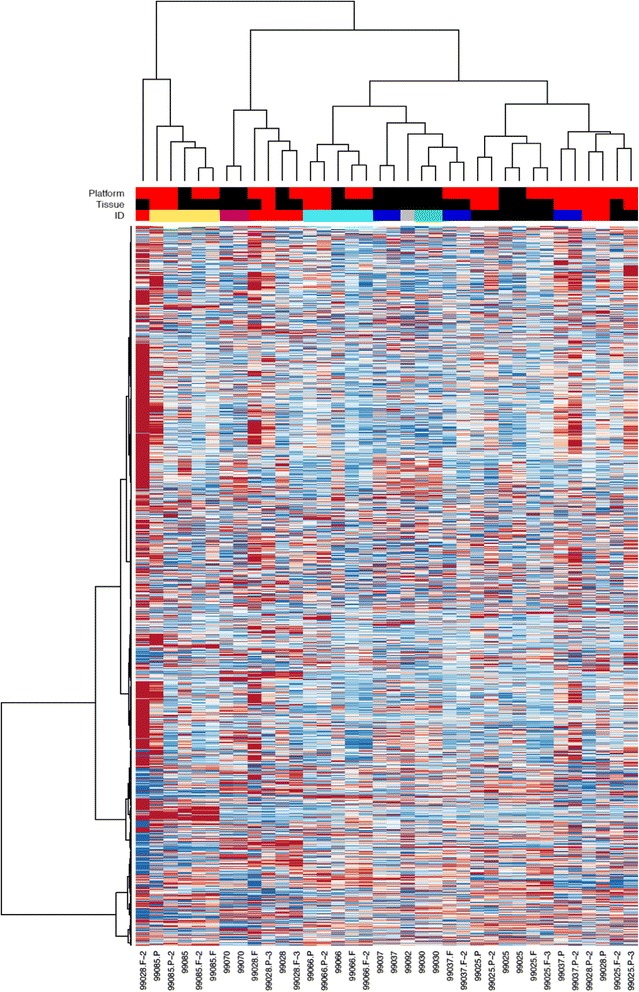
Hierarchical clustering of FF and FFPE samples assessed using the DASL WG assay, and of FF samples assessed using the Illumina BeadArray. This shows significant clustering of FF and FFPE samples, regardless of whether the DASL WG or the BeadArray (indicated by number only) are used

When the analysis was repeated using only the best performing probes (as described in Methods) matched FF and FFPE samples clustered together (Fig. 4). This is one of the first studies to show robustly this signficant overlap using DASL and BeadArray. Four out of the five tumours in which gene expression was assessed using both illumina BeadArray and DASL WG clustered together.

Gene Ontology analaysis was undertaken to assess the probes that were discorant in the FF and FFPE samples to get a better understanding of the biological processes involved (Supplementary File 1). This showed that as far as the biological procesess are concerned: primary metabolic process and nucleic acide metabolic process were the two top in this category. While Poly (A) RNA binding and hetercyclic RNA binging were the top two molecular functions enriched in this group.

## Discussion

This study shows that reliable expression profiles could be generated from FFPE core biopsy tissue, and this supports the future investigation of this platform for analysis of multiple markers in breast cancer. The ability to use FFPE material makes large collections of currently unutilised resources available for molecular marker discovery. Over the past few years, there has been considerable interest in using FFPE tissue for translational research, following the success of the success of the *Recurrence score*® and the PAM50 score, which have demonstrated that reliable tests can be performed using FFPE tissue and used in routine clinical practice [15, 16]. This has led to improvements in the methods used for RNA extraction and in platforms used for FFPE analysis. Multiple tests have been developed for breast, lung, prostate, and colorectal cancer, which can potentially use FFPE samples for molecular marker analysis [17–19].

This is one of the first studies to assess matched FF and FFPE tissues with two different gene expression platforms, and to analyse the concordance of data generated. If progress is to be made with the analysis of FFPE tissue, it is imperative that the limitations of the platforms are investigated. Our study showed that comparable profiles and biological information could be derived.

In a similar study to this, April et al. reported that > 88 % of probes were reliably detected using RNA from both sources, which is comparable to our results [7]. The quality and quantity of RNA that can be extracted from FFPE tissue varies [20]. There are many reasons for these variations, including RNase degradation and genomic DNA contamination [21]. Bibikova et al*.* [6] demonstrated that only 50 % of probes were detected in FFPE compared to FF tumour specimens using DASL CP; however, there were differences between this and the DASL WG array which could account for these differences [22–24]. Linton et al. used Affymetrix Plus 2.0 arrays and found that only 50 % of the probes detected in FF samples were also identified using RNA from FFPE specimens [25]. This is not surprising as the probes in this array are not specifically designed for FFPE analysis and the assay only uses oligo-dT for reverse transcription [26].

Our study carried out a rigorous bioinformatics assessment of the WG DASL platform to validate its role in assessing FFPE samples. The confounding factors such as the GC content and the probe annealing position on the gene are some of the important factors that should be considered in the data analysis to select appropriate probes for comparison. Retrospectively, we also noted that some failed arrays had lower dye incorporation rates and lower total DNA concentration following the PCR reaction. This can potentially be used as a quality control step in the future to improve the data quality. It was also noted that genes that were shorter were more likely to be reliably detected than longer ones. This is going to have implication for selecting the optimal probes for future array design.

Ravo et al*.* analysed the gene expression of matched FF and FFPE samples on a DASL CP platform and also of FF samples on an Illumina BeadArray [27]. Their data suggest that DASL CP is more sensitive than the BeadArray, especially in the identification of low abundance transcripts, due to the gene-specific RT-PCR process [27]. These two assays have different dynamic ranges, which also likely contributes to the differences observed. Similar to this study, where matched RNA was profiled using DASL WG, Mittempergher et al. also found that profiled samples clustered according to the RNA source [11].

One of the unique features of our study, compared to previous reports, is that we used matched FF and FFPE samples and assessed them using the same platform (DASL WG). This enabled us to compare the assay and its performance according to RNA source. Other studies, such as that of Ravo et al., used FFPE and matched cell line samples, rather than FF cancer specimens [27]. April et al. used cancer and matched normal tissue [7], and Wardell et al. analysed samples using different assays [9]. Others such as Sadi et al. compared limited number of probes and Abramovitz et al. compared custom panel of genes, limiting the value in assessing performance [1, 19].

Development of robust bioinformatics methods for analysis and interpretation of FFPE data is needed due to the variable quality of RNA [28]. The close clustering of two types of samples shows that reliable data can be generated using FFPE samples, with significant overlap with profiles from FF samples and thus attesting to the power of this technology to obtain reliable profiles from FFPE tissue. Our data confirm those of Mittempergher et al., which showed that samples profiled using DASL WG clustered together, irrespective of the source of RNA. They also observed that hierarchical clustering of the initial data led to FF and FFPE clustering in groups; however, following median centering of the log_2_ transformed data and use of only the 5444 best performing probes, samples clustered according to type [11]. Our study confirms these results in and a second platform was also used to compare results from FF specimens, adding further evidence of the robustness of the assay.

This confirms the potential of the DASL WG platform for FFPE analysis. Many groups have reported that correlations are poorer for genes with low expression levels. This is of interest as the DASL assay relies on gene-specific amplification; therefore, the signal would be expected to be independent of the expression level. However, it is possible that cDNA synthesis and PCR are less efficient for genes expressed at low levels, especially when modification of the 3′ UTR could cause marked effects.

## Conclusions

Together with appropriate histopathological criteria, molecular classification of cancers can contribute to better patient stratification. This is one of the first studies to assess the DASL WG assay, and importantly to include a comparison with Illumina BeadArray’s. The data demonstrate that reproducible, biologically relevant signatures can be generated using FFPE specimens and the DASL WG assay. This has marked implications for future research using FFPE materials and will impact studies aimed at molecular marker discovery, especially where only FFPE is available.

## Abbreviations

Ct, cycle threshold; DASL cDNA-mediated, annealing, selection, extension, and ligation; FF, fresh frozen; FFPE, formalin-fixed paraffin-embedded; WG, whole-genome
